# Protection or susceptibility to devastating childhood epilepsy: Nodding Syndrome associates with immunogenetic fingerprints in the HLA binding groove

**DOI:** 10.1371/journal.pntd.0008436

**Published:** 2020-07-08

**Authors:** Gil Benedek, Mahmoud Abed El Latif, Keren Miller, Mila Rivkin, Ally Ahmed Ramadhan Lasu, Lul P. Riek, Richard Lako, Shimon Edvardson, Sagit-Arbel Alon, Eithan Galun, Mia Levite

**Affiliations:** 1 Tissue Typing and Immunogenetics Laboratory, Department of Genetics, Hadassah Hebrew University Hospital, Jerusalem, Israel; 2 Goldyne Savad Institute of Gene Therapy, Hadassah Hebrew University Hospital, Jerusalem, Israel; 3 Public Health Consultant, Juba, Republic of South Sudan; 4 External Coordination & Research, Ministry of Health, Juba, Republic of South Sudan; 5 Ministry of Health South Sudan, Juba, Republic of South Sudan; 6 Department of Pediatrics, Neurology Unit, Hadassah Hebrew University Hospital, Jerusalem, Israel; 7 Department of Obstetrics and Gynecology, Hadassah Hebrew University Hospital, Jerusalem, Israel; 8 Faculty of Medicine, The Hebrew University, Jerusalem, Israel; University Hospital Bonn, GERMANY

## Abstract

Nodding syndrome (NS) is a devastating and enigmatic childhood epilepsy. NS is accompanied by multiple neurological impairments and neuroinflammation, and associated with the parasite Onchocerca volvulus (Ov) and other environmental factors. Moreover, NS seems to be an ‘Autoimmune Epilepsy’ since: **1**. ~50% of NS patients have neurotoxic cross-reactive Ov/Leimodin-I autoimmune antibodies. **2**. Our recently published findings: Most (~86%) of NS patients have glutamate-receptor AMPA-GluR3**B** peptide autoimmune antibodies that bind, induce Reactive Oxygen Species, and kill both neural cells and T cells. Furthermore, NS patient’s IgG induce seizures, brain multiple damage alike occurring in brains of NS patients, and elevation of T cells and activated microglia and astrocytes, in brains of normal mice. Human Leukocyte antigen (HLA) class I and II molecules are critical for initiating effective beneficial immunity against foreign microorganisms and contributing to proper brain function, but also predispose to detrimental autoimmunity against self-peptides. We analyzed seven HLA loci, either by next-generation-sequencing or Sequence-Specific-Oligonucleotide-Probe, in 48 NS patients and 51 healthy controls from South Sudan. We discovered that NS associates significantly with both protective HLA haplotype: HLA-B*42:01, C*17:01, DRB1*03:02, DQB1*04:02 and DQA1*04:01, and susceptible motif: Ala24, Glu63 and Phe67, in the HLA-B peptide-binding groove. These amino acids create a hydrophobic and sterically closed peptide-binding HLA pocket, favoring proline residue. Our findings suggest that immunogenetic fingerprints in HLA peptide-binding grooves tentatively associate with protection or susceptibility to NS. Accordingly, different HLA molecules may explain why under similar environmental factors, only some children, within the same families, tribes and districts, develop NS, while others do not.

## Author summary

Nodding syndrome (NS) is a devastating and mysterious neurological disorder affecting 5–15 years old children, primarily in Sudan, Uganda and Tanzania. NS strongly associates with an infection with the parasitic worm Oncocherca Volvulus (Ov), transmitted by the black fly, affecting many people worldwide. Moreover, NS is most probably an 'Autoimmune Epilepsy', especially in view of our recent findings that NS patient’s autoimmune GluR3**B** antibodies induce ROS and kill both neural cells and T cells. NS patient’s IgG also induce seizures, multiple brain damage and inflammation-inducing cells in the brain. HLA class I genes are expressed on the surface of all nucleated cells and present peptides to cytotoxic CD8^+^ T cells. HLA class II genes are expressed mainly on the surface of antigen presenting cells and present peptides to helper CD4+ T cells. Analysis of HLA of South-Sudanese NS patients and healthy controls revealed that that few amino acids in HLA peptide-binding grooves associate with either protection or susceptibility to NS. Theses amino acids could be critical in NS by affecting beneficial immunity and/or detrimental autoimmunity.

## Introduction

Nodding syndrome (NS) is a devastating childhood epilepsy, characterized by severe attacks of nodding of the head, progressive cognitive dysfunction, neurological deterioration, stunted growth and additional pathological neurological features [1–10].

So far, NS was documented primarily in few African countries: South Sudan, Uganda, and Tanzania [4, 9, 10]. Typically, NS affects 5–15 years old children in both sexes. The head nodding episodes are often in association with eating. NS frequently leads to death, typically from secondary causes, after few years from the onset of the disease [2–5]. Despite numerous extensive investigations in the three most NS-affected countries, and outside, the definite primary cause of NS, and the subsequent pathological mechanisms leading to all the different neuropathological features in this disease, are still mysterious.

Having said that, a kaleidoscope of studies over the years revealed various potential causes, features and/or associations of NS with infections, environmental factors, psychological/psychiatric factors, neurodegeneration, and autoimmunity affecting the central nervous system (CNS) [1, 5, 9, 11–14].

Multiple studies show strong association of NS with infection by Onchocerca volvulus (Ov)–a parasitic worm transmitted to humans by the female blackflies of the genus Simulium [15–17].

In fact, Onchocerciasis (River blindness) is a leading neglected tropical disease that affects millions of people in Africa, Latin America and Asia, and causes morbidity, disability, low productivity and poverty in the endemic areas [18, 19]. In the last few years, a wide family of Onhocerca Associated Epilepsy (OAEs) have been revealed, supporting a causative role of Ov in epilepsy too [20–22].

Yet, while Ov seems indeed to contribute to the onset of NS, by itself it cannot account for the entire pathological scenario of NS, primarily since there is still only limited evidence that the parasite itself invades the CNS, and since the infection cannot explain individual differences in the severity and progression of NS within children in the same geographical region and under similar environmental influence.

Various studies revealed potential causes and/or associations of NS with: infections, environmental, psychological/psychiatric factors and neurodegeneration [1, 5, 9, 11–14].

In addition to all these factors, autoimmunity in NS was revealed so far in two studies: 1. by Johnson et al. which discovered that some NS patients have autoimmune antibodies that recognize both an Ov antigen and a self-peptide of Leiomodin-I. These cross-reactive anti-Leiomodin-I/Ov antibodies were detected in 52% of NS patients, as well as in 30.9% of unaffected controls from the same villages, and were demonstrated to be neurotoxic [23]. 2. In a very recently published paper by ourselves [24], we studied 30 South Sudanese NS patients and healthy control subjects, and revealed autoimmune antibodies to 3 extracellular peptides of glutamate receptors (GluR) in NS patients: AMPA-GluR3**B**-peptide antibodies (86%), NMDA-NR1-peptide antibodies (77%) and NMDA-NR2-peptide antibodies (87%). Of these 3 GluR antibodies, NS patient’s affinity-purified AMPA-GluR3**B** were found to have on their own tantalizing pathological effects in vitro: they bind, induce rapid production of ROS in, and then kill both human neural cells and human immune T cells within 1 hour only. Moreover, and most important: In vivo, when NS patient’s purified IgG antibodies were released continuously in the brains of normal mice for 1 week, they induced epileptic seizures and multiple brain damage alike documented in brains of NS patients.

What triggers the production of pathogenic GluR3**B** peptide autoimmune antibodies, and also of the NMDA- NR1 and NR2 antibodies- that are also known to be highly detrimental antibodies-, in NS patients and many patients with other types of epilepsy in which GluR3**B** peptide antibodies are found (for review see [25]), is still unknown.

Regardless of the primary cause of the autoimmunity and NS, the critical fact leading us to the present study is the huge mystery: why does NS affect only some of the children in the very same affected zones, tribes and even families, while other remain healthy, despite the fact they were all most probably exposed to the same environmental factors and infectious organisms.

This led us to hypothesize that immunogenetics in general, and different individual’s specific human leukocyte antigen (HLA) class I and II haplotypes in particular, can underlie the mystery and predispose each individual to either susceptibility to, or rather protection from, this disease.

HLA class I molecules are expressed on all nucleated cells and present intracellular antigens to CD8^+^ cytotoxic T cells [26, 27]. In addition, it is important to note that HLA class I molecules are expressed by subsets of neurons in both the adult and developing mammalian brain (for review, see [28]), and found to play a role in synaptic plasticity, brain development and axonal regeneration [29, 30]. HLA class II molecules are expressed on Antigen Presenting Cells (APCs), which present extracellular antigens to CD4^+^ helper T cells [26, 27]. The HLA loci are one of the most polymorphic loci in humans. The HLA alleles and haplotypes are distributed differently around the world in different ethnic groups [31]. Very few studies evaluated the HLA association with Ov-related diseases [32–34], the first one being that of Meyer et al. that reported that HLA-DQB1*03:01 and DQA1*05:01 are more frequent in individuals putatively resistant to Ov infection [33]. However, the associations of HLA class I and II alleles with NS was yet studied.

In order to comprehensively study the immunogenetics of NS, we analyzed seven HLA loci, by the next-generation sequencing (NGS) method and Sequence Specific Oligonucleotide Probe (SSOP) method, in 48 patients and 51 healthy controls from South Sudan. We found that NS is negatively and significantly associated with HLA-B*42:01, C*17:01, DRB1*03:02, DQB1*04:02 and DQA1*04:01 alleles. This protection could be linked to epitopes at key positions in the respective HLA peptide-binding grooves. In contrast, the disease susceptibility is significantly associated with other defined epitopes at the B pocket of HLA-B peptide-binding groove, and not with specific alleles.

## Methods

### Ethics statement

The study was authorized by: an IRB approval by the Ministry of Health of South-Sudan, related research approval letters, and informed consents of all the NS patients and healthy subjects. In addition a parent or guardian of study participant provided informed consent on the child's behalf.

### Study participants

The study was performed on South Sudanese NS patients (48) (Table 1) and healthy control subjects (51), from the town of Mundri and surrounding villages (a 250 km range area), in Western Equatoria, South-Sudan.

**Table 1:** Clinical characteristics of the South-Sudanese NS patients and healthy controls included in this study.

	NS Patients (n = 48)	Healthy Controls (n = 51)
**Gender**		
Female/male (% females)	22/26 (54)	38/13 (75)
**Age at recruitment**		
Mean (years) ± S.D	14.3±4.0	14.8±5.9
Range	4–25	4–25
**Age at initial NS symptoms**		
Mean (years) ± S.D	8.0±2.9	
Range	4–14	
Nodding with food	37/48	
Nodding with other triggers	11/48	
Other seizures	36/48	
Cognitive decline	38/48	
Low muscle mass	31/34	
Wasting	31/34	

NS was diagnosed and confirmed in all patients included in our study by a pediatric neurologist. In addition, a more comprehensive clinical evaluation was performed for some of them (Table 1). Most of these NS patients had also Generalized Clonic Tonic Seizures (GCTC), on top of the Nodding attacks, as well as various additional pathological symptoms. Healthy South Sudanese subjects, at similar age range, ratio between males and females, and geographical locations to that of the NS patients, were recruited as controls.

Small volume of blood was drawn from all the study subjects by the Sudanese clinicians, and all samples were shipped to Israel for the HLA investigation. Thus, all the studies described in this manuscript were performed in Israel.

Final important note: to the best of our knowledge, the NS patients and the healthy controls whose blood was investigated in this study were NOT tested for Ov infection before the withdrawal of blood for this study. Moreover, we did NOT receive any later information, update, follow-up or blood again, from the same patients and healthy subject since the onset of the study.

### HLA typing

High molecular weight DNA was extracted from blood samples using the MagNA Pure Lc DNA isolation kit, with the automatic device MagNA Pure (Roche).

DNA samples were typed for six loci: HLA-A, -B, -C, -DPB1, -DQB1 and DRB1, using the MX6-1 NGS typing kit (GenDx) according to the manufacturer's instructions. Briefly, the six loci, HLA-A, B, C (whole gene), HLA-DRB1 (exon 2 to exon 3), HLA-DQB1 (exon 2 to part of exon 4) and HLA-DPB1 (exon 2 to exon 5) were amplified by long range PCR. The PCR consists of an initial denaturation step for 3 min at 95°C, and then 25 cycles of the following steps: Denaturation: 15sec at 95°C; Annealing: 30sec at 65°C; Elongation 5 min at, 68°C; Final elongation: 10 min at 68°C. Amplicons were processed in the NGSgo workflow for Illumina using NGSgo-LibrX and NGSgo-IndX (GenDx). The library was quantified using the Qubit dsDNA BR Assay Kit (ThermoFisher Scientific, Waltham, MA, USA) with the Qubit Fluorometer. The sample was denatured with sodium hydroxide, and sequenced at a final concentration of 12pM. The library was paired-end sequenced (2x150bp) on a MiSeq platform (Illumina). FASTQ files were analyzed in NGSengine HLA typing software V2.13 (GenDx), using IPD-IMGT/HLA database 3.33.0. Final genotyping calls were made after manual review.

Further analysis for HLA DQA1 intermediate resolution was performed by the PCR- Sequence Specific Oligonucleotide Probe (SSOP) method of Immucor, life codes [35].

### Statistical analysis

Global Frequencies of *HLA* alleles and an omnibus test for association with specific amino acids were computed using the BIGDAWG R package [36]. Case–control comparisons were then performed by Pearson's *χ*^2^-test, or exact tests as appropriate, if a cell with a count of less than 5 was present. For specific alleles 2 × 2 Pearson's *χ*^2^-test or Fisher's exact test was computed. *P*-values lower than 0.05 were considered statistically significant after applying the Bonferroni correction (*P*_corr_) according to the number of alleles per loci (HLA-B: corrected for 39 tests, HLA-C: corrected for 22 tests, HLA-DRB1: corrected for 18 tests, HLA-DQA1: corrected for 8 tests, HLA-DQB1: corrected for 14 tests and HLA-DPB1 corrected for 10 tests). The odds ratio (OR) and 95% confidence intervals (CI) were calculated for statistically significant alleles, using asymptotic confidence limits, or exact confidence limits when Fisher's exact test was used. When necessary, Haldane's modification was applied. The amino acid frequency was analyzed similarly. *P*-values lower than 0.05 were considered statistically significant after applying the Bonferroni correction (*P*_corr_) according to the number of amino acids per position of the observed alleles (HLA-B positions- 11: corrected for 2 tests, 24: corrected for 3 tests, 63: corrected for 2 tests, 67: corrected for 5 tests. HLA-C positions- 156: corrected for 6 tests, 163: corrected for 3 tests. HLA-DRB1 positions- 71: corrected for 4 tests, 73: corrected for 2 tests, 74: corrected for 5 tests and 77: corrected for 2 tests. HLA-DQA1 position- 69: corrected for 3 tests. HLA-DQB1 positions- 56: corrected for 2 tests, 66: corrected for 2 tests, 67: corrected for 2 tests, 70: corrected for 3 tests and 71: corrected for 4 test). Loci Linkage disequilibrium between different loci was calculated using the HIV molecular immunology database software. The deviation from Hardy-Weinberg equilibrium was not evaluated due to small sample size.

## Results

### NS patients and healthy subjects included in the study

A total of 99 South Sudanese children and young adults: 48 NS patients and 51 healthy controls, aged 4 to 25 years, were recruited for the study, most of them from the same Mundri region, with an approval of the South Sudanese Ministry of Health National IRB. The mean age at initial symptoms was 8 years (SD±2.9). The clinical characteristics and demographic data of the NS patients included in the study are presented in Table 1. Most of the NS patients had also Generalized Tonic Clonic Seizures (GTCS) (75%), cognitive impairment (79%), low muscle mass (91%) and wasting (91%) on top of the nodding attacks. Nodding was frequently triggered by food consumption, immediately following swallowing (77%).

### HLA high and intermediate-resolution genotyping of NS patients and healthy controls

HLA is known to play an important role in both infectious and autoimmune diseases [27]. We tested for significantly different HLA alleles in South Sudanese NS patients vs. South Sudanese healthy controls. There were two specific reasons for this decision: 1. The already reported strong association of NS with infection by Ov; 2. The strong evidences of detrimental anti-CNS autoimmunity in NS, arising from our recent report [24] and the study of Johnson et al [25].

We thus hypothesized that different HLA alleles could lead to either protection or susceptibility to NS, a topic that was not studied so far.

On this basis, HLA typing was performed, by NGS technology for HLA-A, -B, -C, -DRB1, -DQB1 and–DPB1 loci, and by SSOP for HLA-DQA1 locus, for 48 NS patients and 51 healthy controls (S1–S7 Tables). Analysis of HLA class I loci revealed significant differences between the NS patients and healthy controls, in HLA-B and HLA-C (P = 0.04 and P = 0.02, respectively). In contrast, no significant differences were observed in the HLA-A loci. Global analysis of HLA class II loci identified significant differences in HLA-DRB1, DQB1 and DQA1 (P = 0.04, P = 0.01 and P = 0.002, respectively), but not in HLA-DPB1.

As shown in Table 2, HLA-B*15:31 (P nominal = 0.02), HLA-B*35:01 (P nominal = 0.015, OR = 9.18), HLA-C*07:05 (P nominal = 0.025), HLA-DPB1*11:01 (P nominal = 0.005) were associated with susceptibility to NS, but this association did not pass correction for multiple testing, likely due to the small cohort size.

**Table 2:** Frequencies of significantly associated HLA alleles in South Sudanese NS patients and South Sudanese healthy controls.

	Allele frequency				Carrier frequency			
HLA	NS Patients % (N = 48)	Healthy Controls % (N = 51)	P value Corr' (nominal)	OR (95% CI)	NS Patients % (N = 48)	Healthy Controls % (N = 51)	P value Corr' (nominal)	OR (95% CI)
B*15:03	3.13	11.76	(0.02)	0.24 (0.06–0.88)	6.25	21.57	(0.04)	0.24 (0.06–0.93)
B*15:31	5.21	0.00	(0.02)	12.32 ^a^ (0.67–225.04)	10.42	0.00	(0.02)	13.65 ^a^ (0.73–254.07)
B*35:01	8.33	0.98	(0.015)	9.18 (1.12–74.86)	14.58	1.96	(0.02)	8.70 (1.02–73.65)
B*42:01	0.00	9.80	(0.0016)	0**.04** ^**a**^ **(0.002–0.79**)	**0.00**	**19.61**	**0.046**	0**.04** ^**a**^ **(0.002–0.79**)
C*07:05	5.21	0.00	(0.025)	12.32 ^a^ (0.67–225.04)	10.87	0.00	(0.024)	13.65 ^a^ (0.73–254.07)
C*17:01	0.00	10.78	0.015	0**.04** ^**a**^ **(0.002–0.70**)	0.00	21.57	0.011	0**.03** ^**a**^ **(0.002–0.65**)
DRB1*03:02	0.00	10.78	0.012	0**.04** ^**a**^ **(0.002–0.70**)	0.00	21.57	0.009	0**.03** ^**a**^ **(0.002–0.65**)
DQB1*04:02	0.00	10.78	0.009	0**.04** ^**a**^ **(0.002–0.70**)	0.00	21.57	0.007	0**.03** ^**a**^ **(0.002–0.65**)
DQA1*04:01	0.00	10.78	0.005	0**.04** ^**a**^ **(0.002–0.70**)	0.00	21.57	0.004	0**.03** ^**a**^ **(0.002–0.65**)
DPB1*11:01	7.29	0.00	(0.005)	17.17 ^a^ (0.96–305.04)	10.42	0.00	(0.02)	18.61 ^a^ (1.03–335.54)

P-values are presented after the Bonferroni correction or nominal in parentheses for each locus. P, OR and CI values were computed by Pearson’s Chi2 -tests or Fisher’s exact test. a- Haldene's modification

In contrast, HLA-B*15:03 (P nominal = 0.02, OR = 0.24), HLA-B*42:01 (P nominal = 0.0016, OR = 0.04), HLA-C*1701 (Pcorr = 0.015, OR = 0.04), HLA-DRB1*03:02 (Pcorr = 0.012, OR = 0.04), HLA-DQA1*04:01 (Pcorr = 0.006, OR = 0.04) and HLA-DQB1*04:02 (Pcorr = 0.009, OR = 0.04) were associated with protection from NS. Phenotype analysis of carrier frequency revealed similar associations (Table 2).

Taken together, these set of findings suggest that the HLA Class I alleles found here to negatively associate with NS: HLA-B*42:01-C*17:01, which are known to be in strong linkage disequilibrium (LD) (p<0.001), and the HLA class II molecules: DRB1*03:02- DQB1*04:02-DQA1*04:01, which are also in strong LD (P<0.001), are part of the same protective extended haplotype. The alleles of this haplotype, which seem to contribute to protection from NS, are absent in all NS patients, but present in ~10% of the healthy control population.

### Association of HLA amino acid residues with NS

**Fig 1:**
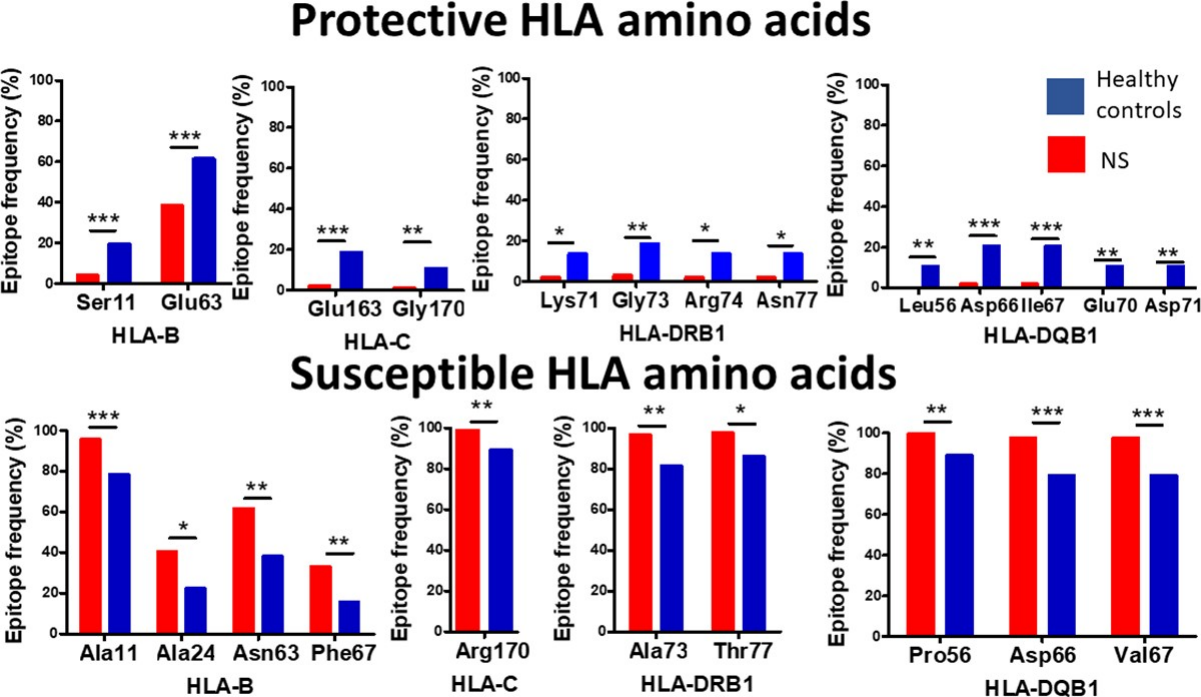
HLA epitopes associated with protection from, or susceptibility to, NS. Association of different epitopes in the HLA binding grooves with NS in South Sudanese patients and South Sudanese healthy controls. Data presented as epitope frequency. *p<0.05, **p<0.01,***P<0.001. Chi^2^ test.

In order to study potential differences in the antigen binding properties of HLA molecules between the NS patients and healthy controls, we examined the association of specific amino acids in the peptide-binding grooves of HLA class I and II molecules, with NS. Omnibus testing revealed that four positions in HLA-B, two positions in HLA-C, three positions in HLA-DRB1, five positions in DQB1, and one position for DQA1, were significantly associated with NS (S8 Table). Of these, as presented in **Fig 1** and S9 Table, in HLA class I the following amino acids were significantly associated with the disease: HLA-B: Aln11 (Pcorr<0.001, OR = 5.61), Aln24 (Pcorr = 0.018, OR = 2.31), Asn63 (Pcorr = 0.002, OR = 2.57) and Phe67 (Pcorr = 0.015, OR = 2.68). This association with susceptibility to NS is driven by the combination of these 3 residues, that are part of B binding pocket of HLA class I (24, 63 and 67), which was observed to be positively associated with NS as well (P = 0.003, OR = 3.25). The statistical significance of this combination that is found in B*35:01, B*51:01 and B*53:01 is greater in comparison to each of these alleles separately (P = 0.015, 0.28 and 0.13, respectively) (S10 Table). In addition, Arg170 in HLA-C mediates disease risk (Pcorr = 0.008, OR = 11.4).

In contrast to these associations, in HLA-B, the presence of Ser in position 11, and Glu in position 63, (Pcorr<0.001 and = 0.002, OR = 0.17 and 0.38, respectively) and in HLA-C -Glu163 (Pcorr<0.001, OR = 0.09), and Gly170 (Pcorr = 0.008, OR = 0.08) were found to have a protective effect.

As for HLA class II molecules, in HLA-DRB1: Ala73 (Pcorr = 0.001, OR = 7.09), Thr77 (Pcorr = 0.005, OR = 7.47) were associated with susceptibility to NS. Whereas, in position 71 Lys (Pcorr = 0.012, OR = 0.13), Gly73 (Pcorr = 0.001, OR = 0.14), were identified as protective. Furthermore, in position 74 that is part of the P4 binding pocket of HLA-DR, our dataset included five different residues in this position, with Arg associated with protection from NS (Pcorr = 0.015, OR = 0.13). Asn77 also significantly associated with protection (Pcorr = 0.005, OR = 0.13). Leu56 and Asp66 (Pcorr = 0.001 and <0.001, OR = 0.16, respectively).

In HLA-DQB1: Pro56 and Glu66 were associated with susceptibility to NS (Pcorr = 0.001 and <0.001, OR = 5.96, respectively). Whereas, Leu56 and Asp66 were found mainly in healthy controls (Pcorr = 0.001 and <0.001, OR = 0.16, respectively). Positions 67 and 70 are part of the P7 binding pocket of HLA-DQ. We found two and three different residues in these positions, respectively. In position 67: Ile was associated with protection from NS (Pcorr<0.001, OR = 0.16). In contrast, Val was associated with susceptibility to NS (Pcorr<0.001, OR = 5.96). However, these two amino acids are both non-polar and hydrophobic. In position 70: Glu was associated with protection from NS (Pcorr = 0.001, OR = 0.04), as this amino acid residue was not observed in any of the NS patients. In addition, Asp in position 71, which was found only in DQB1*04:02, associated disease protection. Finally, in HLA-DQA1: Thr69 (Pcorr = 0.002, OR = 0.04) was also found to associate with protection from NS.

**Fig 2:**
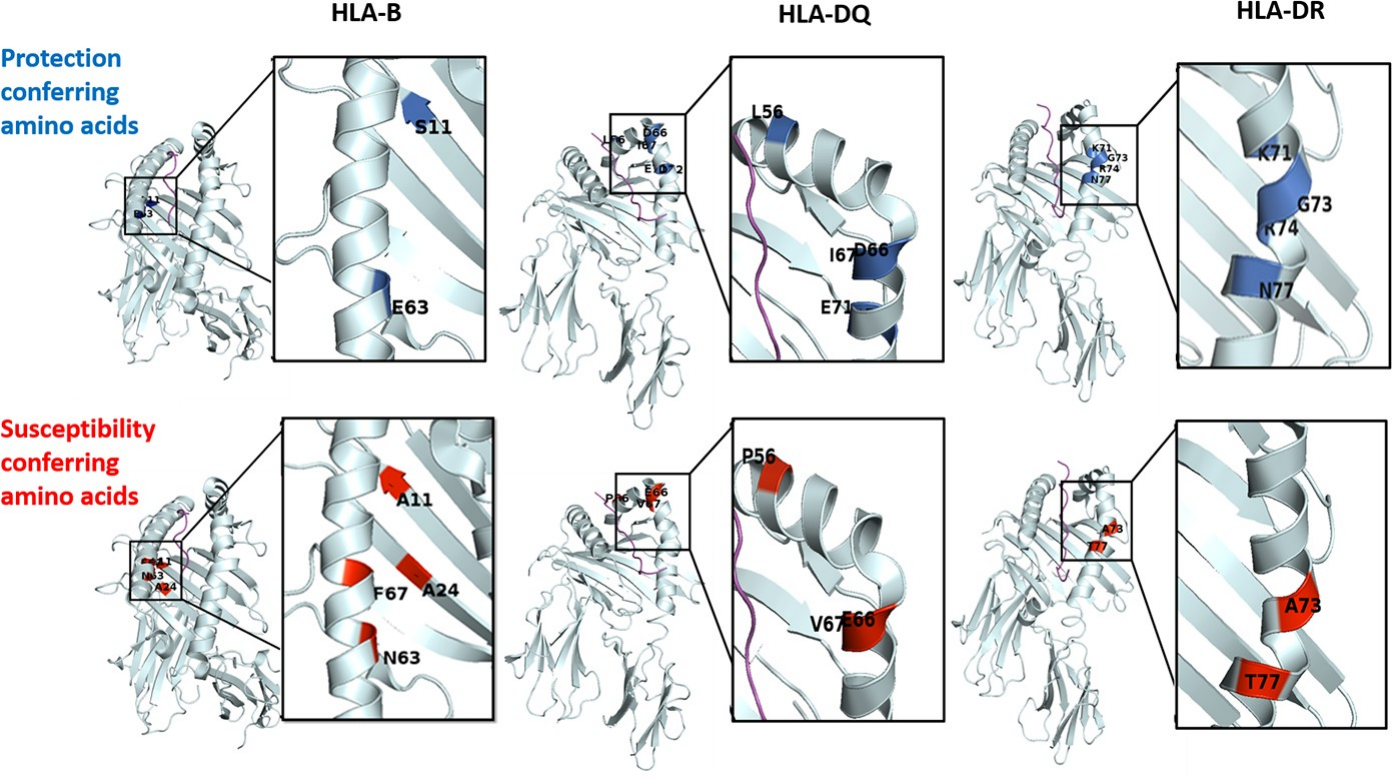
Protective and susceptibility amino acids in HLA class I and class II peptide binding groove. Amino acid positions on HLA-B, DR and DQ peptide binding groove that are associated with protection from (blue) or susceptibility to (red) NS.

Taken together, our data, presented in Figs 1 and 2, suggest that the combination of very few different amino acids located in the HLA class I and II peptide-binding grooves, change the binding affinity of different NS-relevant antigenic peptides to theses grooves, independently of a single specific allele.

## Discussion

NS is a catastrophic neurological disorder affecting 5-15 years old children, characterized by brief pathological nodding seizures, which often begin when the children eat, or sometimes when they feel cold. These seizures are brief and halt after the children stop eating, or when they feel warm again. Often, NS patients also suffer from other types of seizures, mainly GCTS. The children affected by NS experience a complete and permanent stunting of growth. The growth of the brain is also stunted, leading to mental handicap [1–3, 5, 7]. NS is still enigmatic, despite strong association with the parasitic worm Ov [2–4, 10, 19, 22], with other environmental, psychological factors and autoimmunity [6, 8, 23, 24].

Our main findings are that ‘**NS-related immunogenetic fingerprints**’ associate with either susceptibility or protection from the disease and are illustrated graphically in Fig 2.

In summary: in the present study, performed on DNA prepared of blood of NS patients vs. healthy controls from the same region in South Sudan, we discovered, for the first time, that specific amino acids, in HLA-B: Ala11, Ala24, Asn63 and Phe67; DRB1: Ala73, Thr77, and DQB1: Pro56, Glu66 and Val67, located in the respective peptide-binding grooves, associate significantly with susceptibility to NS. In contrast, different amino acids, in HLA-B: Ser11 and Glu63; HLA-C: Trp156 and Glu163; DRB1: Lys71, Gly73, Arg74 and Asn77 and DQB1: Leu56, Asp66, Ile67, Glu70 and Asp71, within the respective peptide-binding grooves, associate with protection from NS.

Thus, we suggest that this ‘amino acids fingerprint’ may explain why only some of the children, within the same geographical region, village and even family, develop NS to begin with, and subsequently all the additional pathological features that follow in the coming years, while others remain healthy.

Structural analysis of the amino acids pattern in the HLA peptide-binding grooves found in this study to associate significantly with either susceptibility to, or protection from NS, revealed interesting features, the first being a protective signature in HLA class II, which is mainly linked to the DRB1*03:02-DQB1*04:02-DQA1*04:01 haplotype. Most of the positions in DRB1 and DQB1, which present epitopes associated with disease susceptibility, were not very polymorphic and contain only two variants. Hence, if one was associated with protection from, the other was associated with susceptibility to, NS. This was mainly evident in the DRB1 locus, with the presence of a hydrophilic amino acid as Gly73 versus a hydrophobic Ala73, and of the positively charged Arg74 at the P4 binding pocket. In our dataset this epitope in P4 was found only in DRB1*03:01 and DRB1*03:02. Interestingly, when evaluating these alleles individually, we did not observe any difference in DRB1*03:01 frequency between NS patients and healthy controls, probably due to its low frequency (<3%). Moreover, in DQB1 66 and 67 positions, either Asp or Glu and Ile or Val were present, respectively. These amino acid pairs have similar properties, but negatively charged amino acids Glu70 and Asp71 were found only in the healthy controls that carry the DQB1*04:02 allele (Fig 1 and S4 Table). It is important to note that most of the DRB3 alleles (although not evaluated in the current study), which could be found on the same haplotypes together with the DRB1*03:01 and DRB1*03:02 alleles, express similar epitopes in positions 71 and 77 (Lys and Asp, respectively). Furthermore, most of the DRB3*01 alleles also express Gly73 and Arg 74. Thus, we cannot rule out the DRB3 contribution to these protective associations.

Our findings allowed us to identify the HLA-B*42:01, C*17:01, DRB1*03:02, DQB1*04:02 and DQA1*04:01 as a protective haplotype in NS, present only in the healthy subjects. Strikingly, none of the 51 NS patients carries this haplotype. Different population studies revealed that while HLA-B*42:01-C*17:01 are found mainly in the presence of DRB1*03:02 and DQB1*04:02-DQA1*04:01, the latter could be linked to different B and C alleles [31]. These findings demonstrate the contribution of the HLA class II to the conferred protection from NS.

Examining the epitopes in the HLA class I peptide-binding groove revealed a significant association of an amino acid signature in the B binding pocket of HLA-B with susceptibility to NS. Interestingly, the frequency Ala24, Glu63 and Phe67 was higher in the NS patients than in the healthy controls. This epitope signature of the B pocket, is present in HLA-B*35:01, *51:01 and *53:01 in our dataset. Together these alleles are part of the B7 supertype alleles, in which the presence of Phe67 creates a hydrophobic, closed-up pocket that can accommodate peptides in an optimal manner, due the presence of proline in position 2 [37, 38].

### HLA in Onchocerciasis

Interestingly, a previous study by De-Angelis et al. on HLA in Ov infection, found that HLA-DQA1*04:01 associates significantly with protection from Onchocerciasis in Ecuadorian populations (Cayapas and Afro-Ecuadorians) [32]. This allele was shown to be in LD with DQB1*04:02. Two other studies on HLA in Ov infections in Bong County, Liberia and in Kaduna State, Northern Nigeria, found that both River blindness and cutaneous features of Onchocerciasis (not "classical" NS) were associated with HLA-DQB1*03:01 and DQA1*05:01 [33, 34]. These alleles were found to be more frequent in individuals putatively immune to Ov infection. In the present study on South Sudanese subjects, we did not find these HLA associations. This might be due to the differences between NS and other types of Onchocerciasis, to different HLA frequencies in these ethnicities, and to the possibility that the NS-related immunogenetic fingerprint’ relates to other immune responses than those against Ov, that affect the development of NS, especially since we cannot determine whether the cohort that was evaluated in this study was infected by Ov. It is also possible that different alleles would be associated with NS in different populations. Thus, it is important to state that while Ov association with NS might be debatable, our findings address HLA associations with NS, and by no mean HLA and Ov infection.

### Possible mechanisms by which HLA could be involved in NS

We postulate that different HLA molecules are important in NS since they can have paramount influence on two critical processes/abilities: 1. Presentation of antigens of infectious organisms by APCS to T cells, in both the periphery and/or CNS, to raise an effective beneficial T cell-mediated immune response to the infectious organism–in the case of NS it is most probably against the parasite Ov; 2. The ability of the individual’s immune system to avoid deterioration from essential and beneficial immune responses against Ov, to detrimental autoimmune responses against self-antigens in the nervous system, and other body systems. In this regard, clear and solid proofs for autoimmune epilepsy in NS were revealed in the work of Johnson et al [23], detecting cross-reactive anti-Leiomodin-I/Ov antibodies that harm neural cells. In addition, our own recent study [24], revealing that a certain type of anti-glutamate receptor antibodies, i.e. GluR3B antibodies of NS patients, bind and kill both human neural cells and normal human T cells by necrosis, and induce seizures and multiple damage in wide areas of the brain, including Cerebellar Purkinje loss and degeneration of the Hippocampus and the Cerebral cortex.

If we assume that Ov is indeed a risk factor in NS, as stated by multiple studies so far [1, 3, 4, 7, 20, 21, 23], we further hypothesize based on our findings that both CD4^+^ and CD8^+^ T cells might be involved in the pathogenesis of NS. On the one hand, the known interaction of specific HLA class II molecules with helper CD4^+^ T cells could induce beneficial protection against Ov infection, Onchocerciasis and NS. On the other hand, the known interaction of specific HLA class I molecules with cytotoxic CD8^+^ T cells could contribute to detrimental autoimmunity in Onchocerciasis, OAE and NS.

In this regard, since we found herein that HLA class I are associated with the disease, we also suggest that CD8^+^ T cells are important for the beneficial eradication of Ov in NS, not only to the detrimental autoimmunity. However, the exact role of CD8^+^ T cells in NS awaits further elucidation.

Finally, in line with our study, a functional role of HLA alleles in autoimmune diseases was recently shown in a model of Goodpasture disease. Ooi et al. demonstrated that the presentation of the same autoreactive peptide by susceptibility or protective alleles, could lead to different T cells responses [39].

Having suggested this mechanism, it is important to note that as in other autoimmune diseases or infectious diseases, there are other factors, in addition to the HLA, that affect disease protection or susceptibility. Furthermore, as in such diseases, in which HLA is associated with protection, the protective alleles are carried by minority of the total population. Similarly, not all affected individuals carry the susceptibility alleles, but the frequency is significantly higher compared with healthy subjects [40–42].

As to the cohort size and the geographical zone of the patients and controls, four issues are noteworthy. First, needless to say, that our findings call for, and will hopefully stimulate, larger scale immunogenetic studies on many more NS patients in different countries, and on other forms of OAE.

Second, we had great difficulties in recruiting South Sudanese NS patients and healthy subjects to this study, due to several reasons, primarily the active ongoing war. As such, recruiting 48 NS patients and 51 healthy subjects was an achievement, although the study could benefit from more.

Third, it is important to note that although our cohort of 48 NS patients could be seen as a relatively small one, the observed HLA allele frequencies in the South Sudanese healthy control group are similar to the frequencies reported for healthy subjects in neighboring populations, such as in Kenya [43, 44]. Interestingly, the DRB1*03:02-DQB1*04:02-DQA1*04:01 haplotype, which is more common in African populations, was reported to be a protective against an autoimmune disease- namely Type 1 diabetes in African Americans [40], suggesting that this haplotype might be associated with general protection against several autoimmune diseases.

Forth, it is possible that subsequent studies, on other NS-affected populations, would reveal that other HLA alleles and other amino acids within their peptide-binding groove than those found herein significantly associate with NS, and confer either susceptibility or protection from the disease. Nevertheless, such possible future findings are not expected to change the novelty of our current discovery on the role of HLA in NS.

In conclusion, we discovered a unique amino acid signature in HLA peptide-binding grooves that might affect both immunity and autoimmune processes in NS in South-Sudan. We identified both a protective HLA haplotype and a susceptible structural motif that are associated with the disease. The novel immunogenetic findings of our study may explain why under the same environmental factors, only some children, within the same families, tribes and districts, develop this malady while others can prevent or overcome it and therefore remain healthy, and may shed new light on other epileptic disorders and Onchocerciasis.

## Supporting information

S1 Table HLA-A frequencies in South Sudanese NS patients and South Sudanese healthy controls. (DOCX)

S2 Table HLA-B frequencies in South Sudanese NS patients and South Sudanese healthy controls. (DOCX)

S3 Table HLA-C frequencies in South Sudanese NS patients and South Sudanese healthy controls. (DOCX)

S4 Table HLA-DRB1 frequencies in South Sudanese NS patients and South Sudanese healthy controls. (DOCX)

S5 Table HLA-DQB1 frequencies in South Sudanese NS patients and South Sudanese healthy controls. (DOCX)

S6 Table HLA-DQA1 frequencies in South Sudanese NS patients and South Sudanese healthy controls. (DOCX)

S7 Table HLA-DPB1 frequencies in South Sudanese NS patients and South Sudanese healthy controls. (DOCX)

S8 Table Significant amino acid positions that are different between NS patients and South Sudanese healthy controls. (DOCX)

S9 Table HLA epitopes associated with protection from, or susceptibility to, NS. (DOCX)

S10 Table HLA-B alleles with Ala24, Glu63 and Phe67. (DOCX)
