# Supplementary material for: Protection or susceptibility to devastating childhood epilepsy: Nodding Syndrome associates with immunogenetic fingerprints in the HLA binding groove
Source: PLoS Negl Trop Dis. 2020 Jul 8;14(7):e0008436. doi: 10.1371/journal.pntd.0008436 (PMC7371228; doi:10.1371/journal.pntd.0008436)
Supplement: S1 Table — (DOCX) [file pntd.0008436.s001.docx]

**Table S1: HLA-A frequencies in South Sudanese NS patients and South Sudanese healthy controls**

| **OR**  **(95% CI)** | **P value (nominal)** | **Healthy Controls % (2N=102)** | **NS**  **Patients %**  **(2N=96)** | **HLA-A*** |
| --- | --- | --- | --- | --- |
|  |  | 3.92 | 4.17 | **01:01** |
|  |  | 9.80 | 11.46 | **02:01** |
|  |  | 2.94 | 1.04 | **02:02** |
|  |  | 2.94 | 0.00 | **02:05** |
|  |  | 4.90 | 9.38 | **03:01** |
|  |  | 0.98 | 0.00 | **05:02** |
|  |  | 0.00 | 1.04 | **13:02** |
|  |  | 7.84 | 6.25 | **23:01** |
|  |  | 6.86 | 10.42 | **23:17** |
|  |  | 0.98 | 0.00 | **24:02** |
|  |  | 3.92 | 0.00 | **26:12** |
|  |  | 3.92 | 0.00 | **29:01** |
|  |  | 0.00 | 4.17 | **29:02** |
|  |  | 7.84 | 2.08 | **30:01** |
|  |  | 1.96 | 3.13 | **30:02** |
|  |  | 1.96 | 5.21 | **30:04** |
|  |  | 0.98 | 3.13 | **31:01** |
|  |  | 5.88 | 5.21 | **31:04** |
|  |  | 1.96 | 4.17 | **32:01** |
|  |  | 2.94 | 2.08 | **33:01** |
|  |  | 1.96 | 0.00 | **33:03** |
|  |  | 9.80 | 4.17 | **34:02** |
|  |  | 1.96 | 1.04 | **36:01** |
|  |  | 0.00 | 1.04 | **38:01** |
|  |  | 0.98 | 1.04 | **66:01** |
|  |  | 5.88 | 1.04 | **68:01** |
|  |  | 3.92 | 10.42 | **68:02** |
|  |  | 1.96 | 3.13 | **74:01** |
|  |  | 0.00 | 4.17 | **74:03** |
|  |  | 0.98 | 1.04 | **32:106** |
