# Supplementary material for: Protection or susceptibility to devastating childhood epilepsy: Nodding Syndrome associates with immunogenetic fingerprints in the HLA binding groove
Source: PLoS Negl Trop Dis. 2020 Jul 8;14(7):e0008436. doi: 10.1371/journal.pntd.0008436 (PMC7371228; doi:10.1371/journal.pntd.0008436)
Supplement: S2 Table — (DOCX) [file pntd.0008436.s002.docx]

**Table S2: HLA-B frequencies in South Sudanese NS patients and South Sudanese healthy controls**

| **OR (95% CI)** | **P value (nominal)** | **Healthy Controls % (2N=102)** | **NS**  **Patients %**  **(2N=96)** | **HLA-B*** |
| --- | --- | --- | --- | --- |
|  |  | 1.04 | 0.00 | **07:02** |
|  |  | 0.00 | 0.98 | **07:05** |
|  |  | 9.38 | 6.86 | **08:01** |
|  |  | 9.38 | 17.65 | **13:02** |
|  |  | 3.13 | 0.00 | **14:02** |
|  |  | 4.17 | 0.00 | **14:03** |
| 0.24 (0.06-0.88) | (0.02) | 3.13 | 11.76 | **15:03** |
|  |  | 2.08 | 0.98 | **15:10** |
|  |  | 1.04 | 0.00 | **15:16** |
|  |  | 2.08 | 0.00 | **15:18** |
| 12.32 ^a^ (0.67-225.04) | (0.02) | 5.21 | 0.00 | **15:31** |
|  |  | 1.04 | 0.98 | **15:55** |
|  |  | 0.00 | 0.98 | **15:65** |
|  |  | 0.00 | 1.96 | **18:01** |
|  |  | 0.00 | 0.98 | **27:03** |
| 9.18 (1.12-74.86) | (0.015) | 8.33 | 0.98 | **35:01** |
|  |  | 1.04 | 0.00 | **39:02** |
|  |  | 1.04 | 1.96 | **39:10** |
|  |  | 0.00 | 0.98 | **39:24** |
|  |  | 1.04 | 0.98 | **40:12** |
|  |  | 2.08 | 0.98 | **41:01** |
|  |  | 1.04 | 0.98 | **41:02** |
| 0.04 ^a^ (0.002-0.79) | (0.0016) | 0.00 | 9.80 | **42:01** |
|  |  | 0.00 | 1.96 | **42:02** |
|  |  | 1.04 | 0.00 | **44:03** |
|  |  | 2.08 | 2.94 | **45:01** |
|  |  | 2.08 | 0.98 | **47:01** |
|  |  | 4.17 | 5.88 | **47:03** |
|  |  | 3.13 | 0.98 | **51:01** |
|  |  | 12.50 | 6.86 | **53:01** |
|  |  | 1.04 | 1.96 | **57:01** |
|  |  | 0.00 | 0.98 | **57:02** |
|  |  | 1.04 | 0.00 | **57:03** |
|  |  | 7.29 | 8.82 | **58:01** |
|  |  | 1.04 | 0.00 | **73:01** |
|  |  | 0.00 | 0.98 | **81:01** |
|  |  | 7.29 | 1.96 | **82:02** |
|  |  | 0 | 0.98 | **15:151** |
|  |  | 1 | 4.90 | **15:220** |

P-values are presented after the Bonferroni correction (corrected for 39 tests), or as nominal P-values in parentheses. P, OR and CI values shown are from Pearson’s Chi2 -tests except for the HLA-B*15:31 and B*42:01 alleles, which were computed by Fisher’s exact test. a- Haldene's modification
