# Supplementary material for: Protection or susceptibility to devastating childhood epilepsy: Nodding Syndrome associates with immunogenetic fingerprints in the HLA binding groove
Source: PLoS Negl Trop Dis. 2020 Jul 8;14(7):e0008436. doi: 10.1371/journal.pntd.0008436 (PMC7371228; doi:10.1371/journal.pntd.0008436)
Supplement: S3 Table — (DOCX) [file pntd.0008436.s003.docx]

**Table S3: HLA-C frequencies in South Sudanese NS patients and South Sudanese healthy controls**

| **OR (95% CI)** | **P value (nominal)** | **Healthy Controls % (2N=102)** | **NS Patients % (2N=96)** | **HLA-C*** |
| --- | --- | --- | --- | --- |
|  |  | 1.96 | 0 | **02:02** |
|  |  | 5.88 | 1.04 | **02:10** |
|  |  | 3.92 | 8.33 | **03:02** |
|  |  | 1.96 | 1.04 | **03:04** |
|  |  | 11.76 | 16.67 | **04:01** |
|  |  | 0.98 | 1.04 | **04:04** |
|  |  | 0.98 | 4.17 | **04:07** |
|  |  | 13.73 | 7.29 | **06:02** |
|  |  | 4.90 | 5.21 | **07:01** |
|  |  | 4.90 | 9.38 | **07:02** |
|  |  | 0.98 | 1.04 | **07:04** |
| 12.32 ^a^(0.67-225.04) | (0.025) | 0.00 | 5.21 | **07:05** |
|  |  | 15.69 | 12.50 | **07:18** |
|  |  | 5.88 | 8.33 | **08:02** |
|  |  | 1.96 | 2.08 | **12:03** |
|  |  | 0.98 | 0.00 | **12:167** |
|  |  | 0.98 | 1.04 | **14:02** |
|  |  | 0.98 | 1.04 | **15:05** |
|  |  | 4.90 | 5.21 | **16:01** |
| 0.04 ^a^(0.002-0.70) | 0.015 | 10.78 | 0.00 | **17:01** |
|  |  | 0.00 | 1.04 | **17:03** |
|  |  | 5.88 | 8.33 | **18:02** |

P-values are presented after the Bonferroni correction (corrected for 22 tests), or as nominal P-values in parentheses. P, OR and CI values shown are from Pearson’s Chi2 -tests except for the HLA-C*07:05 and C*17:01 alleles, which were computed by Fisher’s exact test. a- Haldene's modification.
