# Supplementary material for: Protection or susceptibility to devastating childhood epilepsy: Nodding Syndrome associates with immunogenetic fingerprints in the HLA binding groove
Source: PLoS Negl Trop Dis. 2020 Jul 8;14(7):e0008436. doi: 10.1371/journal.pntd.0008436 (PMC7371228; doi:10.1371/journal.pntd.0008436)
Supplement: S4 Table — (DOCX) [file pntd.0008436.s004.docx]

**Table S4: HLA-DRB1 frequencies in South Sudanese NS patients and South Sudanese healthy controls**

| OR (95% CI) | P value (nominal) | Healthy Controls % (2N=102) | NS Patients % (2N=96) | **HLA-DRB1*** |
| --- | --- | --- | --- | --- |
|  |  | 3.92 | 4.17 | **01:01** |
|  |  | 5.88 | 6.25 | **01:02** |
|  |  | 2.94 | 2.08 | **03:01** |
| 0.04 ^a^(0.002-0.70) | 0.012 | 10.78 | 0.00 | **03:02** |
|  |  | 3.92 | 2.08 | **04:05** |
|  |  | 4.90 | 1.04 | **07:01** |
|  |  | 1.96 | 3.13 | **08:04** |
|  |  | 1.96 | 1.04 | **09:01** |
|  |  | 0.98 | 4.17 | **10:01** |
|  |  | 16.67 | 21.88 | **11:01** |
|  |  | 8.82 | 14.58 | **11:02** |
|  |  | 0.00 | 1.04 | **11:10** |
|  |  | 4.90 | 1.04 | **12:01** |
|  |  | 2.94 | 2.08 | **13:01** |
|  |  | 9.80 | 10.42 | **13:02** |
|  |  | 0.98 | 2.08 | **13:16** |
|  |  | 0.00 | 1.04 | **14:54** |
|  |  | 18.63 | 21.88 | **15:03** |

P-values are presented after the Bonferroni correction (corrected for 18 tests). P, OR and CI values were computed by Fisher’s exact test. a- Haldene's modification.
