# Supplementary material for: Protection or susceptibility to devastating childhood epilepsy: Nodding Syndrome associates with immunogenetic fingerprints in the HLA binding groove
Source: PLoS Negl Trop Dis. 2020 Jul 8;14(7):e0008436. doi: 10.1371/journal.pntd.0008436 (PMC7371228; doi:10.1371/journal.pntd.0008436)
Supplement: S5 Table — (DOCX) [file pntd.0008436.s005.docx]

**Table S5: HLA-DQB1 frequencies in South Sudanese NS patients and South Sudanese healthy controls**

| **OR (95% CI)** | **P value (nominal)** | **Healthy Controls % (2N=102)** | **NS Patients % (2N=96)** | **HLA-DQB1*** |
| --- | --- | --- | --- | --- |
|  |  | 2.94 | 2.08 | **02:01** |
|  |  | 6.86 | 2.08 | **02:02** |
|  |  | 10.78 | 16.67 | **03:01** |
|  |  | 3.92 | 2.08 | **03:02** |
|  |  | 1.96 | 3.13 | **03:19** |
| 0**.**04 ^a^(0.002-0.70) | 0.009 | 10.78 | 0.00 | **04:02** |
|  |  | 27.45 | 28.13 | **05:01** |
|  |  | 0.00 | 1.04 | **05:02** |
|  |  | 0.00 | 1.04 | **05:03** |
|  |  | 21.57 | 29.17 | **06:02** |
|  |  | 0.98 | 0.00 | **06:03** |
|  |  | 3.92 | 4.17 | **06:04** |
|  |  | 1.96 | 2.08 | **06:08** |
|  |  | 6.86 | 8.33 | **06:09** |

P-values are presented after the Bonferroni correction (corrected for 14 tests). P, OR and CI values were computed by Fisher’s exact test. a- Haldene's modification
