# Supplementary material for: Protection or susceptibility to devastating childhood epilepsy: Nodding Syndrome associates with immunogenetic fingerprints in the HLA binding groove
Source: PLoS Negl Trop Dis. 2020 Jul 8;14(7):e0008436. doi: 10.1371/journal.pntd.0008436 (PMC7371228; doi:10.1371/journal.pntd.0008436)
Supplement: S6 Table — (DOCX) [file pntd.0008436.s006.docx]

**Table S6: HLA-DQA1 frequencies in South Sudanese NS patients and South Sudanese healthy controls**

| **OR (95% CI)** | P value  Corr' (nominal) | **Healthy Controls % (2N=102)** | **NS Patients % (2N=96)** | **HLA-DQA1*** |
| --- | --- | --- | --- | --- |
|  |  | 14.71 | 16.67 | **01:01** |
|  |  | 45.10 | 56.25 | **01:02** |
|  |  | 2.94 | 2.08 | **01:03** |
|  |  | 4.90 | 1.04 | **02:01** |
|  |  | 5.88 | 3.13 | **03:02** |
| 0**.**04 ^a^ (0.002-0.70) | 0.005 | 10.78 | 0.00 | **04:01** |
|  |  | 2.94 | 1.04 | **05:01** |
|  |  | 12.75 | 19.79 | **05:05** |

P-values are presented after the Bonferroni correction (corrected for 8 tests). P, OR and CI values were computed by Fisher’s exact test. a- Haldene's modification.
