# Supplementary material for: Protection or susceptibility to devastating childhood epilepsy: Nodding Syndrome associates with immunogenetic fingerprints in the HLA binding groove
Source: PLoS Negl Trop Dis. 2020 Jul 8;14(7):e0008436. doi: 10.1371/journal.pntd.0008436 (PMC7371228; doi:10.1371/journal.pntd.0008436)
Supplement: S7 Table — (DOCX) [file pntd.0008436.s007.docx]

**Table S7: HLA-DPB1 frequencies in South Sudanese NS patients and South Sudanese healthy controls**

| **OR**  **(95% CI)** | **P value (nominal)** | **Healthy Controls % (2N=102)** | **NS**  **Patients %**  **(2N=96)** | **HLA-DPB1*** |
| --- | --- | --- | --- | --- |
|  |  | 19.61 | 20.83 | **01:01** |
|  |  | 7.84 | 6.25 | **02:01** |
|  |  | 1.96 | 3.13 | **03:01** |
|  |  | 4.90 | 8.33 | **04:01** |
| 17.17 ^a^ (0.96-305.04) | (0.005) | 0.00 | 7.29 | **11:01** |
|  |  | 4.90 | 9.38 | **13:01** |
|  |  | 6.86 | 2.08 | **17:01** |
|  |  | 3.92 | 0.00 | **18:01** |
|  |  | 0.98 | 0.00 | **30:01** |
|  |  | 1.96 | 1.04 | **39:01** |
|  |  | 0.98 | 0.00 | **40:01** |
|  |  | 0.00 | 1.04 | **417:01** |
|  |  | 0.00 | 1.04 | **461:01** |
|  |  | 1.96 | 0.00 | **49:01** |
|  |  | 0.98 | 0.00 | **61:01N** |
|  |  | 0.00 | 2.08 | **99:01** |
|  |  | 7.84 | 2.08 | **104:01** |
|  |  | 22.55 | 17.71 | **105:01** |
|  |  | 0.98 | 0.00 | **107:01** |
|  |  | 0.00 | 1.04 | **654:01** |
|  |  | 11.76 | 16.67 | **665:01** |

P-values are presented as nominal P-values in parentheses. P, OR and CI values were computed by Fisher’s exact test. a- Haldene's modification.
