# Supplementary material for: Protection or susceptibility to devastating childhood epilepsy: Nodding Syndrome associates with immunogenetic fingerprints in the HLA binding groove
Source: PLoS Negl Trop Dis. 2020 Jul 8;14(7):e0008436. doi: 10.1371/journal.pntd.0008436 (PMC7371228; doi:10.1371/journal.pntd.0008436)
Supplement: S8 Table — (DOCX) [file pntd.0008436.s008.docx]

**Table S8: Significant amino acid positions that are different between NS patients and South Sudanese healthy controls**

| **HLA** | **Position** | **P value** |
| --- | --- | --- |
| **HLA-B** | 11 | 0.001 |
|  | 24 | 0.02 |
|  | 63 | 0.001 |
|  | 67 | 0.001 |
| **HLA-C** | 163 | 0.001 |
|  | 170 | 0.01 |
| **HLA-**  **DRB1** | 71 | 0.02 |
|  | 73 | 0.001 |
|  | 74 | 0.007 |
|  | 77 | 0.006 |
| **HLA-DQB1** | 56 | 0.002 |
|  | 66 | 0.001 |
|  | 67 | 0.001 |
|  | 70 | 0.003 |
|  | 71 | 0.002 |
| **HLA-DQA1** | 69 | 0.003 |

Analysis of significantly different epitopes between NS and HC subjects in HLA class I and II.
