# Supplementary material for: Protection or susceptibility to devastating childhood epilepsy: Nodding Syndrome associates with immunogenetic fingerprints in the HLA binding groove
Source: PLoS Negl Trop Dis. 2020 Jul 8;14(7):e0008436. doi: 10.1371/journal.pntd.0008436 (PMC7371228; doi:10.1371/journal.pntd.0008436)
Supplement: S9 Table — (DOCX) [file pntd.0008436.s009.docx]

**Table S9:** **HLA epitopes associated with protection from, or susceptibility to, NS**

| **HLA** | **Position** | **Epitope** | **NS Patients (2n=96)** | **Healthy Controls (2n=102)** | **P value corrected** | **OR**  **(CI 95%)** | **Associated alleles** |
| --- | --- | --- | --- | --- | --- | --- | --- |
| **Class I**  **HLA-B** | 11 | Ser | 4.2% | 19.6% | <0.001 | 0.17 (0.05-0.54) | *07:02, 07:05, 18:01, 27:03, 39:02, 39:10, 39:24, 42,01, 42:02, 73:01, 81:01 |
|  |  | Ala | 95.8% | 78.4% | <0.001 | 5.61 (1.84-17.09) | *08:01, 13:02, 14:02, 15:03, 15:10, 15:16, 15:18, 15:31, 15:55, 15:65, 15:151, 15:220, 35:01, 40:12, 41:01, 41:02, 44:03, 45:01, 47:01, 47:03, 51:01, 53:01, 57:01, 57:03, 58:01, 82:02 |
|  | 24 | Ala | 40.6% | 22.5% | 0.018 | 2.31 (1.26-4.35) | *08:01, 13:02, 14:02, 15:03, 15:10, 15:16, 15:18, 15:31, 15:55, 15:65, 15:151, 15:220, 35:01, 40:12, 41:01, 41:02, 44:03, 45:01, 47:01, 47:03, 51:01, 53:01, 57:01, 57:03, 58:01, 82:02 |
|  | 63 | Glu | 38.2% | 61.5% | 0.002 | 0.38 (0.21-0.68) | *07:02, 07:05, 14:02, 14:03, 15:10, 15:18, 15:31, 15:55, 18:01, 35:01, 39:10, 39:24, 42,01, 42:02,51:01, 53,:01, 73:01, 81:01, 82:02 |
|  |  | Asn | 61.8% | 38.5%% | 0.002 | 2.57 (1.45-4.56) | *13:02, 15:03, 15:16, 15:65, 15:151, 15:220, 27:03, 39:02, 40:12, 41:01, 41:02, 44:03, 45:01, 47:01, 47:03, 57:01, 57:02, 57:03, 58:01 |
|  | 67 | Phe | 33.3% | 15.7% | 0.015 | 2.68 (1.35-5.31) | *08:01, 35:01, 51:01, 53:01 |
| **Class I**  **HLA-C** | 163 | Glu | 2.1% | 18.6% | <0.001 | 0.09 (0.02-0.41) | *02:02, 02:10, 17:01, 17:03 |
|  | 170 | Gly | 1.1% | 10.8% | 0.004 | 0.08 (0.01-0.68) | *17:01, 17:03 |
|  |  | Arg | 98.9% | 89.2% | 0.004 | 11.4 (1.45-90.7) | *02:02, 02:10, 03:02, 03:04, 04:01, 04:04, 04:07, 06:02, 07:01, 07:02, 07:04, 07:05, 07:18, 08:02, 12:03, 12:167, 14:02, 15:05, 16:01, 18:02 |
| **Class II**  **HLA-**  **DRB1** | 71 | Lys | 2.1% | 13.7% | 0.012 | 0.13 (0.02-0.60) | *03:01, 03:02 |
|  | 73 | Gly | 3.1% | 18.6% | 0.001 | 0.14 (0.04-0.49) | *03:01, 03:02, 07:01 |
|  |  | Ala | 96.9% | 81.4% | 0.001 | 7.09 (2.02-24.84) | *01:01,01:02, 04:05, 08:04, 09:01, 10:01, 11:01, 11:02, 12:01, 13:01, 13:02, 13:16, 14:54, 15:03 |
|  | 74 | Arg | 2.1% | 13.7% | 0.015 | 0.13 (0.02-0.60) | *03:01, 03:02 |
|  | 77 | Asn | 2.1% | 13.7% | 0.005 | 0.13 (0.02-0.60) | *03:01, 03:02 |
|  |  | Thr | 97.9% | 86.3% | 0.005 | 7.47 (1.65-33.8) | *01:01,01:02, 04:05, 07:01, 08:04, 09:01, 10:01, 11:01, 11:02, 12:01, 13:01, 13:02, 13:16, 14:54, 15:03 |
| **Class II**  **HLA-DQB1** | 56 | Leu | 0 | 10.8% | 0.001 | 0.04 ^a^ (0.002-0.70) | *04:02 |
|  |  | Pro | 100% | 89.2% | 0.001 | 24.25 ^a^ (1.40-417.6) | *02:01, 02:02, 03:01, 03:02, 03:19, 05:01, 05:03, 06:02, 06:03, 06:04, 06:08, 06:09 |
|  | 66 | Asp | 2.1% | 20.6% | <0.001 | 0.16 (0.05-0.50) | *02:01, 02:02, 04:02 |
|  |  | Glu | 97.9% | 79.4% | <0.001 | 5.96 (1.96-18.09) | *03:01, 03:02, 03:19, 05:01, 05:03, 06:02, 06:03, 06:04, 06:08, 06:09 |
|  | 67 | Ile | 2.1% | 20.6% | <0.001 | 0.16 (0.05-0.50) | *02:01, 02:02, 04:02 |
|  |  | Val | 97.9% | 79.4% | <0.001 | 5.96 (1.96-18.09) | *03:01, 03:02, 03:19, 05:01, 05:03, 06:02, 06:03, 06:04, 06:08, 06:09 |
|  | 70 | Glu | 0 | 10.8% | 0.002 | 0.04 ^a^ (0.002-0.70) | *04:02 |
|  | 71 | Asp | 0 | 10.8% | 0.001 | 0.04 ^a^ (0.002-0.70) | *04:02 |
| **Class II**  **HLA-DQA1** | 69 | Thr | 0 | 10.8% | 0.002 | 0.04^a^ (0.002-0.70) | *04:01 |

P-values are presented after the Bonferroni correction. OR and CI values shown are from Pearson’s Chi2 –tests. a- Haldene's modification.
