# Supplementary material for: Protection or susceptibility to devastating childhood epilepsy: Nodding Syndrome associates with immunogenetic fingerprints in the HLA binding groove
Source: PLoS Negl Trop Dis. 2020 Jul 8;14(7):e0008436. doi: 10.1371/journal.pntd.0008436 (PMC7371228; doi:10.1371/journal.pntd.0008436)
Supplement: S10 Table — (DOCX) [file pntd.0008436.s010.docx]

**Table S10: HLA-B alleles with Ala24, Glu63 and Phe67**

| **OR**  **(95% CI)** | **P value** | **Healthy Controls % (2N=102)** | **NS**  **Patients %**  **(2N=96)** | **HLA-B*** |
| --- | --- | --- | --- | --- |
| 9.18 (1.12-74.86) | 0.015 | 0.98 | 8.33 | **35:01** |
| 3.25 (0.33-34.87) | 0.28 | 0.98 | 3.13 | **51:01** |
| 1.93 (0.72-5.15) | 0.13 | 6.86 | 12.5 | **53:01** |
| 3.25 (1.42-7.46) | 0.003 | 8.82 | 23.96 | **Combined** |

P-values are presented as nominal. P, OR and CI values were computed by Fisher’s exact test.
